# Elucidating the Signal Responses of Multi-Parametric Surface Plasmon Resonance Living Cell Sensing: A Comparison between Optical Modeling and Drug–MDCKII Cell Interaction Measurements

**DOI:** 10.1371/journal.pone.0072192

**Published:** 2013-08-27

**Authors:** Tapani Viitala, Niko Granqvist, Susanna Hallila, Manuela Raviña, Marjo Yliperttula

**Affiliations:** 1 Division of Biopharmaceutics and Pharmacokinetics, Faculty of Pharmacy, University of Helsinki, Helsinki, Finland; 2 Centre for Drug Research, Faculty of Pharmacy, University of Helsinki, Helsinki, Finland; Centro Nacional de Biotecnologia – CSIC, Spain

## Abstract

*In vitro* cell-based assays are widely used during the drug discovery and development process to test the biological activity of new drugs. Most of the commonly used cell-based assays, however, lack the ability to measure in real-time or under dynamic conditions (e.g. constant flow). In this study a multi-parameter surface plasmon resonance approach in combination with living cell sensing has been utilized for monitoring drug-cell interactions in real-time, under constant flow and without labels. The multi-parameter surface plasmon resonance approach, i.e. surface plasmon resonance angle versus intensity plots, provided fully specific signal patterns for various cell behaviors when stimulating cells with drugs that use para- and transcellular absorption routes. Simulated full surface plasmon resonance angular spectra of cell monolayers were compared with actual surface plasmon resonance measurements performed with MDCKII cell monolayers in order to better understand the origin of the surface plasmon resonance signal responses during drug stimulation of cells. The comparison of the simulated and measured surface plasmon resonance responses allowed to better understand and provide plausible explanations for the type of cellular changes, e.g. morphological or mass redistribution in cells, that were induced in the MDCKII cell monolayers during drug stimulation, and consequently to differentiate between the type and modes of drug actions. The multi-parameter surface plasmon resonance approach presented in this study lays the foundation for developing new types of cell-based tools for life science research, which should contribute to an improved mechanistic understanding of the type and contribution of different drug transport routes on drug absorption.

## Introduction

Current drug discovery paradigms are slowly shifting from the reductionism thinking approach towards a more holistic approach [1], [2]. The ability to examine living cells in physiologically relevant environments, to monitor drug induced cell stimuli, and differentiating between different drug delivery routes are of utmost importance for improving our mechanistic understanding during the drug discovery and development processes [2]–[5]. Therefore, cell-based assays have gained increased popularity compared to biochemical assays in drug discovery and development. Although cell-based assays are more complex and less specific than biochemical assays, they facilitate the measurements of mode of action, pathway activation, toxicity, and phenotypic responses of cells mediated by exogenous stimuli. However, established *in vitro* cell-based assays are static and laborious and cannot measure real-time interactions on the cellular level. They often rely on labeled materials for imaging or detection purposes, and they require a secondary detection technique where the final quantification is based on UV- or fluorescence spectroscopy, mass spectrometry, radiometry or chromatographic techniques. Thus, a development of new *in vitro* cell-based assay methodologies and approaches which enable direct detection, and real-time, non-invasive, label-free and continuous high sensitivity monitoring of cell responses to exogenous stimuli, would be desirable.

Several label-free techniques have recently been developed for studying cell-substrate adhesion, cell-cell interactions, cell migration and volume changes in cells [6]–[14], as well as for monitoring living cell activity (e.g. cellular metabolism, toxicity, receptor mediated signaling and endocytic vesicle formation) [15]–[26]. Among the label-free techniques developed for probing the activities and interactions of living cells, optical techniques that utilizes evanescent waves, i.e. surface plasmon resonance (SPR) and resonant waveguide grating (RWG), have attracted a great deal of interest. This is probably because they are widely spread and have established themselves as powerful techniques for biosensing applications. However, the evanescent wave measuring techniques generally penetrate approximately ½ of the incident light wavelength into the surrounding medium. Thus, for a visible light source, a 300 nm penetration depth with an exponential decay of sensitivity as a function of distance from the sensor surface is commonly achieved [27]. This means that in living cell sensing, the evanescent wave technique only probes the bottom part of the cell layer. Attempts to improve the penetration depth have been made by utilizing near infrared (NIR) SPR [12], [24], but despite of this the active scanning range is still well below the common cell diameter.

An advantage of SPR compared to RWG is that SPR systems are capable of measuring in constant and controlled flow conditions, and depending on the optical setup of the SPR instrument, it is even possible to extract thickness and refractive index information on the (cell) layers through optical modeling of the full SPR spectrum [27], [28]. SPR has established itself as a powerful technique for providing affinity and kinetic information of target-based biomolecular interactions [29], [30]. However, several studies have demonstrated that SPR is also a powerful tool for real-time monitoring of living cell interactions, and for studying different cellular processes without the use of labeling agents [15], [16], [18], [19]–[22], [24], [26]., So far all SPR interaction studies with living cells are performed by measuring and analyzing only changes either in the main SPR peak angular position or in the reflection intensity at a fixed angle near the main SPR peak minimum. This probably origins from one or both of the following reasons: 1) Traditionally SPR has almost solely been used for routine biomolecular interaction analysis based on reflectance or angular changes, and the living cell sensing is therefore forced into the same thinking patterns, and/or 2) the SPR instruments used for living cell studies do not provide any other information than reflection intensity at fixed angle or angular change information, which does not allow for any other type of analysis.

The full SPR angular spectra have successfully been used in modeling optical properties and thicknesses of both thin organic and inorganic layers [27], [28], [31]. However, a highly unexploited approach of SPR is to measure the full SPR angular spectra in real-time in order to fully utilize its shape or key parameters (i.e. SPR peak angular position, SPR peak minimum intensity and the changes in the total internal reflection region) for studying drug interaction processes with cellular targets. This might not be critical when considering traditional biomolecular interactions, but it should play a significant role in living cell sensing. Therefore, analyzing multiple parameters from the full SPR angular spectra would be of interest in order to try to obtain a better quantitative or even qualitative understanding of how SPR could be utilized for living cell sensing. No studies have so far made use of real-time monitoring of the full SPR angular spectra and utilized it for analyzing real-time drug-cell interactions.

Herein, changes in simulated full SPR angular spectra induced by varying different optical parameters are compared with actual SPR measurements of drug-MDCK II cell interactions in order to elucidate the signal responses in living cell sensing with SPR. An understanding of cell-analyte responses is established through optical modeling of different sections of the cell monolayer, and by examining the changes taking place in the full SPR angular spectra caused by the introduction of an analyte. The simulated SPR angular spectra responses from a cell monolayer are then compared with the measured full SPR angular spectra of an actual cell monolayer composed of MDCKII cells. Finally, a new qualitative analysis method demonstrating how the multi-parameter SPR approach enables to distinguish between passive (trans- and paracellular) drug absorption processes during drug-cell interactions is presented.

## Experimental Section

### SPR theory

**Figure 1:**
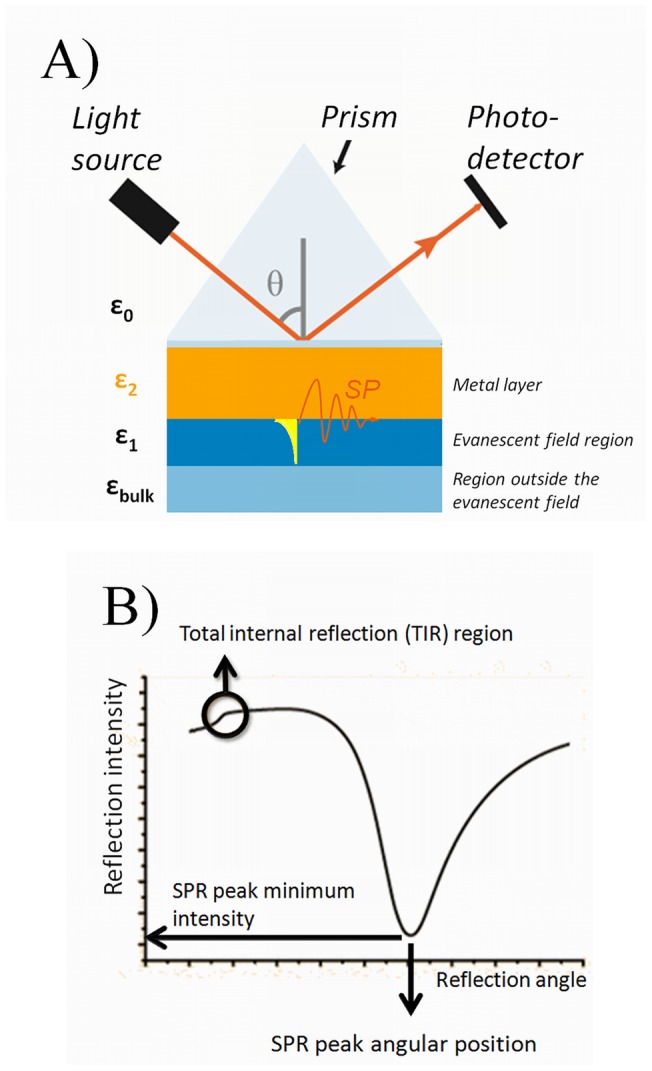
Kretschmann configuration and key parameters obtained from the full SPR angular spectra. A) A simplified chart of the Kretschmann configuration enabling plasmon excitations and SPR measurements. The intensity of the reflected light from a monochromatic light source is measured as a function of incident light angle (θ). The light passes from a high refractive index medium (glass, ε_0_) to a low refractive index medium (air or liquid, ε_1_+ε_bulk_). In between, the light is reflected from an interface containing a metal with a high density of free electrons and an optimal thickness for plasmon excitation (gold 50 nm, ε_2_) to a photodetector. The surface plasmons on the metal surface are excited at a certain incident light angle (θ) and the evanescent field created by the plasmon extends to the adjacent low refractive index medium (ε_1_) where samples are introduced to the system. B) A schematic full SPR angular spectrum showing the positions of the TIR region, the main SPR peak angular position and the main SPR peak minimum intensity.

The working principle of the SPR technique is based on utilizing visible light to excite free electrons on a surface of a metal, which in turn causes surface plasmons to travel along the metal surface also creating an evanescent field to the adjacent medium in contact with the metal. The surface plasmon excitation takes place when certain conditions regarding the optical properties of the system and incident light angular frequency are matched, resulting in a high absorption of the incident light. The most common way of fulfilling these conditions is to use the so-called Kretchmann configuration (Figure 1A), which enables the detection of plasmon excitation from a sharp dip in the refracted light intensity (Figure 1B). The total internal reflection (TIR) region is sensitive to the optical properties of the media outside the evanescent field (ε_bulk_), whereas the main SPR peak angular position and intensity are highly sensitive to the optical properties of the media within the evanescent field (ε_1_) [27], [28].

A theoretical mathematical description for the surface plasmon resonance condition for a multilayer optical system can be obtained by solving the Maxwell equations. This results in the following solution for the resonance condition:
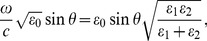
(1)where ω is the angular frequency of light, c is the speed of light, θ is the angle of the incident light and ε_0_, ε_1_ and ε_2_ are the permittivity of the prism, of the SPR metal layer and of the adjacent medium, respectively (Figure 1). The permittivity (ε) and refractive index (ñ) of the materials can be written in their complex forms as following:



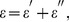
(2a)

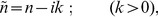
(2b)and the permittivity and the complex refractive index also have the following relationship:



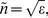
(3a)

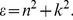
(3b)


A general answer for the Maxwell equations for multilayered systems linked to measurable or controllable variables can be solved by using the transfer matrix formalism of 2×2 matrices. The overall mathematical formalism has been published several times, and it is not in the scope of this article to discuss it in detail [28], [31]. In practice, this matrix formalism is solved by mathematical calculations and fitting tools, or by taking advantage of dedicated software tools developed for this (e.g. Winspall) [32]. Fitting the full SPR angular spectra then provides information that can be used to characterize sample properties, such as the real refractive index, the thickness and the light absorbance properties of different analyst materials at the surface [28], [33], [34].

### Simulation of full SPR angular spectra

The Winspall software (version 3.02) [32] was used throughout this study to simulate the full SPR angular spectra. The optical parameters for the fixed components in the simulations were as following: Glass prism, n = 1.5294, k = 0 and thickness  = ∞; Chromium adhesion layer, n = 3.1085, k = 3.4873 and thickness  = 1.53 nm, Gold layer, n = 0.2262, k = 3.7639 and thickness  = 50.59 nm. The optical parameters for the chromium adhesion and the gold layer were extracted from a Winspall fit to a measured full SPR angular spectrum of a real and thoroughly cleaned gold-coated SPR sensor slide immersed in water. All simulations were done with the sample layer immersed in a bulk medium resembling water with n = 1.3299 and k = 0. The incident light wavelength used in the simulations was 670 nm, which is the same wavelength as in the SPR device used in this study.

### Cell culture

Madin-Darby canine kidney (MDCKII) cells were cultured in Dulbecco's Modified Eagle Medium (D-MEM) (Gibco) supplemented with 10% heat-inactivated FBS and 1% penicillin-streptomycin. Cells were maintained at 37°C in a 5% CO_2_ incubator.

### Immobilization of cells on surface plasmon resonance sensor

Gold-coated SPR sensor slides were obtained from Bionavis Ltd. (Tampere, Finland). Before the experiments the sensors were first cleaned by boiling them for 5 min in a solution containing 1 part of 30% ammonia hydroxide solution (Sigma), 1 part of 30% hydrogen peroxide (Sigma) in 5 parts of Milli-Q-water. Hereafter, the sensors were rinsed thoroughly with Milli-Q-water and dried with nitrogen. Finally, the SPR sensor slides were autoclaved before cell immobilization.

The immobilization of MDCKII cells on the SPR sensor slides were performed by first treating confluent cell layers in cell culture flasks with 0.25% trypsin/EDTA in DPBS, followed by a re-suspension of the cells in the cell culture medium. The SPR sensor slide was then placed in a cell culturing polystyrene petri dish with a cell growth area of 8.8 cm^2^ and 3 ml of the cell suspension was pipetted on top of the SPR sensor slide. Cells were then allowed to attach and grow on the SPR sensor slide in an incubator in a controlled environment until they were confluent.

### Viability of cells on SPR sensor slides

The trypan blue test was performed on cells cultured both in tissue culture treated polystyrene wells as a reference, and on cells cultured directly on SPR sensor slides. After 24 h of culturing the medium was carefully removed, and the cells were washed with DPBS before detaching them with a 0,25% trypsin/EDTA solution. Cells were then resuspended in cell culture medium and a Trypan blue solution (Gibco) was added to the cell suspension in a ratio of 1∶1 (v:v) in order to stain the dead cells blue. The non-colored cells (viable cells) were counted with a Cedex XS cell counter (Roche Diagnostics Oy).

### Test compounds in living cell surface plasmon resonance analysis

Propranolol hydrochloride (Sigma-Aldrich) and D-mannitol (Fluka) were used as test compounds in the SPR interaction studies with living cells. Each test compound was diluted in a buffer composed of Hank's Balanced Salt Solution (HBSS, Gibco) supplemented with 10 mM Hepes (Sigma) and adjusted to pH 7.4 with 1 M NaOH (running buffer).

### Surface plasmon resonance analysis

Interaction experiments between test compounds and immobilized MDCKII cells were performed using a multiparameter SPR device (MP-SPR Navi 200, BioNavis Ltd, Tampere, Finland). The cells were cultured on the SPR sensor slide for 3–4 days before analysis. Just before the measurements, the whole flow path of the SPR device was filled with the running buffer. Once the cells had reached confluency on the SPR sensor slide, they were once washed with the running buffer. After this, the sensor slide was quickly inserted into the instrument before the cell layer could dry. The experiments were performed under a constant flow rate of 10 μl/min, which was controlled by a syringe pump accessory. The interaction between cells and test compounds were measured by injecting the compound of interest for 6 to 10 min followed by a rinsing period of 10–20 minutes with pure running buffer. All the experiments were performed at 20 °C by using the angular scan mode. The angular scan range during the experiments was between 60–78°. With this scan range the angular scan mode provided a full SPR angular spectrum every four seconds. At the end of each experiment, the SPR sensor slide was examined under an optical microscope in order to evaluate the cell monolayer integrity after the interaction experiments.

## Results

### Immobilization of MDCKII cell monolayers on SPR sensor slides

**Figure 2:**
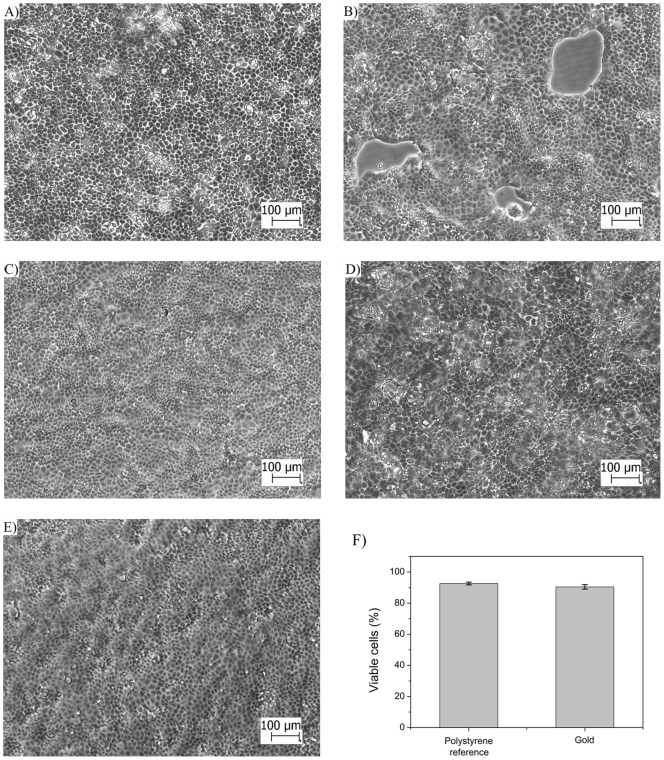
MDCKII cell morphology and viability on polystyrene and gold coated SPR sensor slide. Light microscopy images of MDCKII cells cultured on A) polystyrene with a cell seeding density of 1×10^5^ cells/cm^2^, B) SPR sensor slide with a cell seeding density of 5×10^4^ cells/cm^2^ , C) SPR sensor slide with a cell seeding density of 7×10^4^ cells/cm^2^ , D) SPR sensor slide with a cell seeding density of 1×10^5^ cells/cm^2^, E) SPR sensor slide with a cell seeding density of 7×10^4^ cells/cm^2^ after exposing the cell monolayer to increasing concentration (2.5 μM, 25 µM and 250 µM) of propranolol during 1 hour at a flow rate of 10 μl/min in the SPR flow channel, F) cell viability of MDCKII cells grown on polystyrene reference and gold coated SPR sensor slides. The cell seeding time used for the cell monolayers in A)–F) was 3 days. The scale bar in all images is 100 µm.

**Figure 3:**
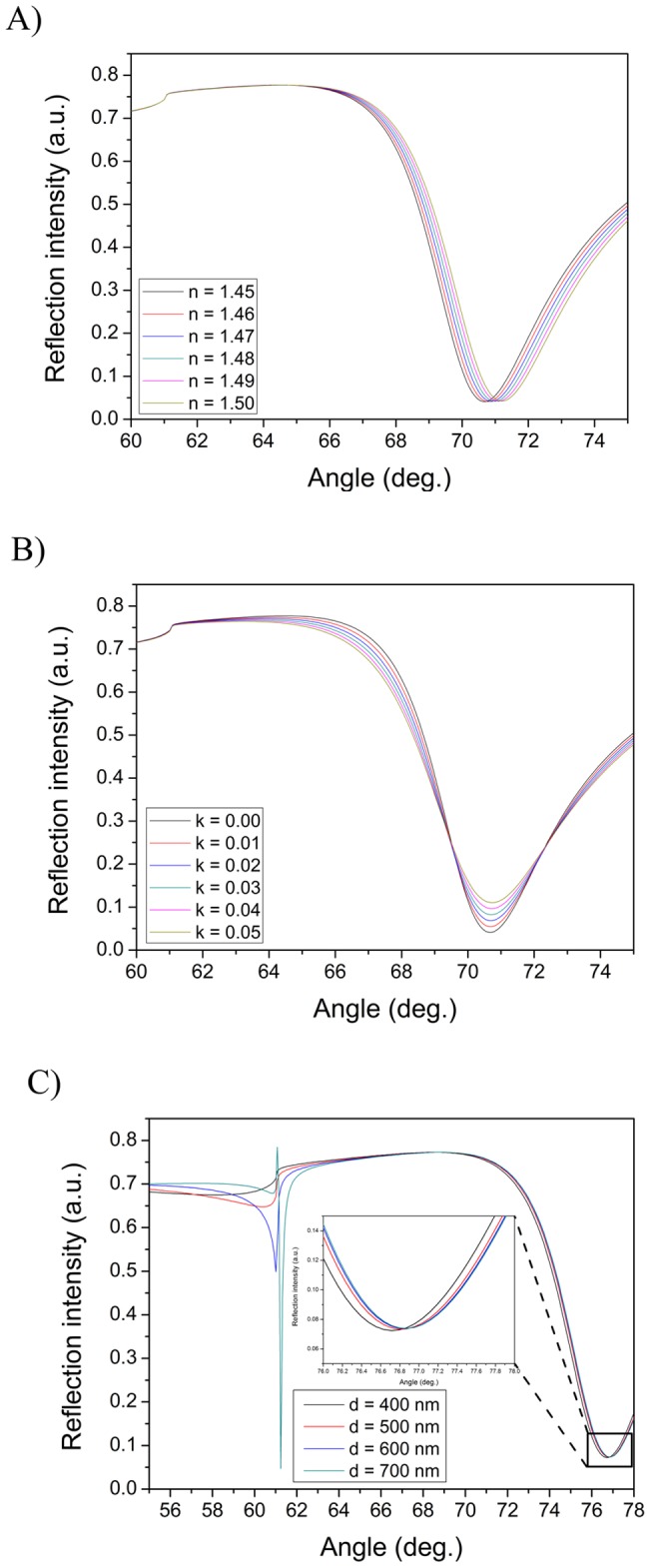
Simulated full SPR spectra for optical changes within the evanescent field and thick sample layers. Behavior of simulated full SPR angular spectra when A) changing the real and B) changing the imaginary parts of the refractive index components, and C) for very thick (waveguide) sample layers. The following parameters were used for simulations: A) sample layer thickness: 10 nm, k = 0 and n varied from 1.45–1.5, B) sample layer thickness: 10 nm, n = 1.45 and k varied from 0.00–0.05, and C) n = 1.38, k = 0 and sample layer thickness varied from 400–700 nm.

Real-time drug-cell interactions were monitored with SPR by immobilizing a monolayer of MDCKII cells on the SPR sensor slide while continuously measuring the full SPR angular spectrum during cell stimulation with propranolol and D-mannitol. For this purpose, it was of utmost importance to optimize the cell immobilization protocol, because the surface coverage of cells on the SPR sensor slide has a dramatic influence on the shape of the full SPR angular spectra [12], [16], [24]. The morphology of MDCKII cells seeded directly on SPR sensor slides and polystyrene surfaces (used as a reference) was found to be the same (Fig. 2A–D). Different cell seeding densities from 5×10^4^ cells/cm^2^ to 1×10^5^ cells/cm^2^ on the SPR sensor slide revealed the following: 1) The lowest cell seeding density of 5×10^4^ cells/cm^2^ was not sufficient to form a fully confluent cell monolayer and large cell free areas could be seen in the microscopy image (Fig. 2B), 2) the highest cell seeding density of 1×10^5^ cells/cm^2^ showed some cell-condensed clusters (Fig. 2D), and 3) an intermediate cell seeding density of 7×10^4^ cells/cm^2^ was optimal for immobilizing a uniform, almost cluster-free and fully confluent cell monolayer on the SPR sensor slide (Fig. 2C). The optimum cell seeding time for achieving confluent MDCKII cell monolayers on SPR sensor slides was also determined to be 3–4 days.

Stimulation experiments with drugs showed that the MDCKII cell monolayers on the SPR sensor slides remained confluent with hardly any changes in morphology after being exposed to the drug at a flow rate of 10 μl/min in the SPR flow channel (Fig. 2E). The flow experiments clearly demonstrated that there is no need to use any adhesion promoter in order to successfully immobilize and retain a confluent MDCKII cell monolayer on the SPR sensor slide for SPR interaction measurements. The trypan blue cell viability test showed that the MDCKII cells remained viable on the SPR sensor slides after 24 h of cell culturing (Fig. 2F).

Thus, the optimized cell seeding conditions allowed to consistently preparing uniform, fully confluent and viable MDCKII cell monolayers on the SPR sensor slides. This ensures that no significant contributions to the SPR signal will be caused by cell spreading, cell division, cluster formation or overgrown cell monolayers.

### Simulated full SPR angular spectra of cell monolayers

In the majority of biomolecular or biosensing interaction studies with SPR, the sample layer thickness is well below the penetration depth of the evanescent field. In such a case, changes in the real part of the refractive index (n) will to a good approximation reflect the mass of the analyte within the sample layer. This is exemplified with the simulated full SPR angular spectra in Figure 3A. This figure shows that the main SPR peak angular position will shift to higher angles when the refractive index (n) of a sample layer increases from 1.45 to 1.5. On the other hand, if n would have been kept constant in the simulation in Figure 3A, then an increase in the sample layer thickness would have caused a corresponding increase in the main SPR peak angular position angle. This is the case, as long as the layer thickness is smaller than the penetration depth of the evanescent field. A much less frequently characterized property when using SPR is the imaginary part of the refractive index (k), which is linked to the absorbance or scattering of light by the sample layer. Figure 3B shows the changes in simulated full SPR angular spectra caused by an increase in k from 0 to 0.05 in the sample layer. It is clear that the change in k induces an increase in the main SPR peak minimum intensity, which means that less of the light used to excite surface plasmons actually can do so. The reason for this is that the sample absorbs or scatters light, which consequently changes the conditions for exciting surface plasmons. In such a case the optical properties of the system start to deviate from the optimum conditions for surface plasmon excitation described by equation (1). This in turn leads to a situation where an increasing amount of light is reflected back instead of exciting the surface plasmons.

Figure 3C shows another exotic optical behavior of the simulated full SPR angular spectra. When the sample layer thickness reaches the corresponding thickness of the penetration depth of the SPR evanescent field, then the main SPR peak angular position is shifted to very high angles and a second peak starts to appear in the vicinity of the total internal reflection (TIR) angle. After this a further increase in the sample layer thickness does not induce any major changes in the main SPR peak angular position, but the second peak in the vicinity of the TIR angle will become more pronounced. This behavior of the full SPR angular spectra is due to the formation of a waveguide on the SPR sensor slide [34], [35]. Figure 3C also reveals that the waveguide peak grows stronger when the layer thickness approaches the wavelength of the light used in the simulation (i.e. 670 nm), or when the sample layer thickness is close to twice the penetration depth of the evanescent field.

**Figure 4:**
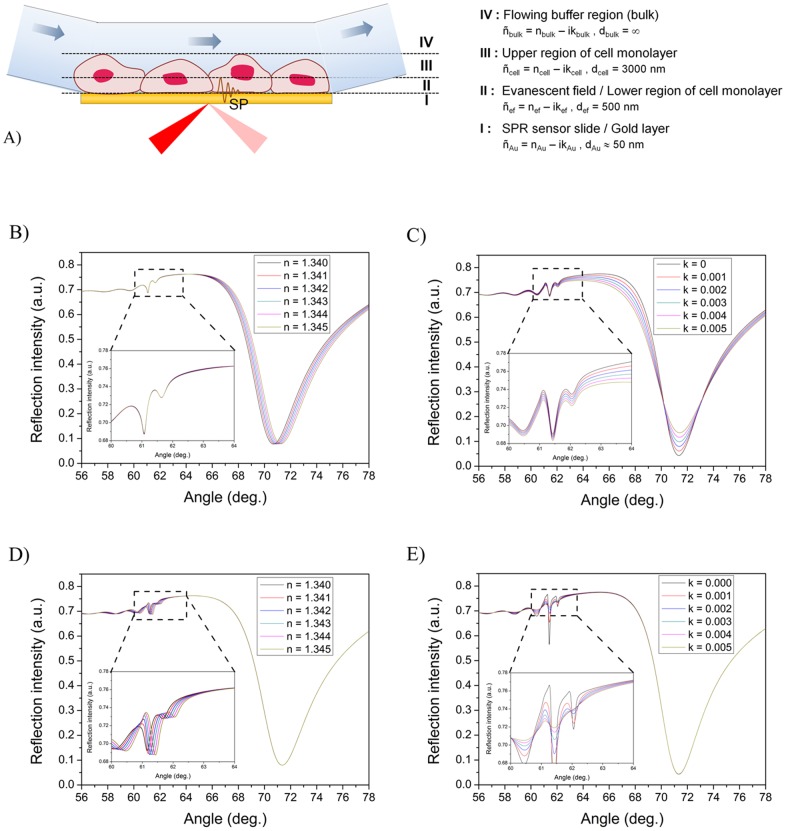
Simulated full SPR spectra for optical changes within different regions of a cell monolayer. A) Schematic representation of the sample layer used in simulating full SPR angular spectra of cell monolayers. The cell monolayer was theoretically split into three sections in order to clarify the effect of changing different optical properties in it: 1) a thin section in the magnitude of evanescent field close to the sensor surface (*ñ_ef_*, *d_ef_*  = 500 nm), 2) a thick section consisting of the rest of the cell (*ñ_cell_*, *d_cell_*  = 3000 nm), and 3) an infinite bulk medium layer (*ñ_bulk_*, *d_bulk_*  =  ∞). Simulated full SPR angular spectra when changing; B) the real (*n_ef_*) and C) the imaginary (*k_ef_*) parts of the refractive index for a cell monolayer within the evanescent field, D) the real part of the refractive index (*n_cell_*) for a cell monolayer not within the evanescent field and E) the imaginary part of the refractive index (*k_cell_*) for a cell monolayer not within the evanescent field. Insets in B-E are more detailed views of the TIR regions. The following parameters were used for simulations: B) *d_cell_*  = 3000 nm, *k_cell_*  = 0.002, *n_cell_* varied from 1.340–1.345, *d_ef_*  = 500 nm, *n_ef_*  = 1.34 and *k_ef_*  = 0.002, C) *d_cell_*  = 3000 nm with *n_cell_*  = 1.340, *k_cell_* varied from 0–0.005, *d_ef_*  = 500 nm, *n_ef_*  = 1.34 and *k_ef_*  = 0.002, D) *d_cell_*  = 3000 nm, *k_cell_*  = 0.002, *n_cell_*  = 1.34, *d_ef_*  = 500 nm, *k_ef_*  = 0.002 and *n_ef_* varied from 1.340–1.345, and E) *d_cell_*  = 3000 nm, *k_cell_*  = 0.002, *n_cell_*  = 1.34, *d_ef_*  = 500 nm, *n_ef_*  = 1.34 and *k_ef_* varied from 0–0.005.

When a cell monolayer is taken as the sample layer in SPR it is expected that the behavior of the full SPR angular spectra should differ dramatically compared to a sample layer with a thickness well below the penetration depth of the evanescent field. The cell monolayer is a very thick water rich layer with a thickness of a few to tens of micrometers, and with a refractive index very close to water. Thus, the thickness of the cell monolayer is much larger than the penetration depth of the evanescent field. Figure 4A shows a schematic representation for the sample layer used in simulating full SPR angular spectra of cell monolayers. In order to clarify the effect of changing different optical properties in a cell monolayer, the cell monolayer is theoretically split into three sections, i.e. a thin section in the magnitude of evanescent field close to the sensor surface (ñ_ef_, 500 nm), a thick section consisting of the rest of the cell (ñ_cell_, 3000 nm), and an infinite bulk medium layer (ñ_bulk_, buffer).

When the full SPR angular spectra of a cell monolayer based on the layer structure in Figure 3A was simulated, it was found that a cell monolayer should form a weak waveguide on the SPR sensor slide (Fig. 4B–E). This is indicated by the small peaks and wavy curves in the TIR region (see Figure 3). An increase in the real part of the refractive index of a cell monolayer (*n_ef_*) within the evanescent field (region III in Fig. 4A) resulted in an increase in the main SPR peak angular position (Fig. 4B), with negligible changes in the main SPR peak minimum intensity and TIR region (inset in Fig. 4B). Similarly, an increase in the complex part of the refractive index (*k_ef_*) induces an increase in the main SPR peak minimum intensity and a ***decrease*** in the intensity around the TIR angle, but no changes in the main SPR peak angular position (Fig. 4C and 4D). It is worth noting that 10 times smaller refractive index changes are needed for the cell monolayer compared to a thin sample layer (see Figure 3) in order to induce the corresponding changes in the main SPR peak angular position or main SPR peak minimum intensity.

If the *n_cell_* for the cell monolayer outside the evanescent field (region II in Fig. 4A) was increased in the simulations, then no changes were seen in the main SPR peak angular position, but the TIR angle increased slightly (Fig. 4D). Correspondingly, when *k_cell_* was increased in the same region, then no changes were seen in the main SPR peak minimum intensity (Fig. 4E). The intensity changes in the TIR region also showed a rather complex behavior with several inflection points at which the intensity changes reversed direction. However, the intensity changes around the TIR angle (∼62°) became negligible when *k_cell_* reached 0.002–0.003 (inset in Fig. 4E) when compared to the intensity changes induced by changes in *k_ef_* within the evanescent field (inset of Figure 4C). Additionally, no changes in the main SPR peak angular position or the SPR peak minimum intensity were found in cases where the complete cell monolayer thickness (*d_ef_ + d_cell_*) was varied between 2000–6000 nm or when only *n_bulk_* of the bulk medium layer was varied between 1.330–1.355 (Figure S1 and S2). In these cases there were also only negligible changes in the TIR region when the cell layer thickness was larger than 3000 nm, which is still much smaller than the actual thickness of a cell.

### Drug-cell interaction analysis with surface plasmon resonance

**Figure 5:**
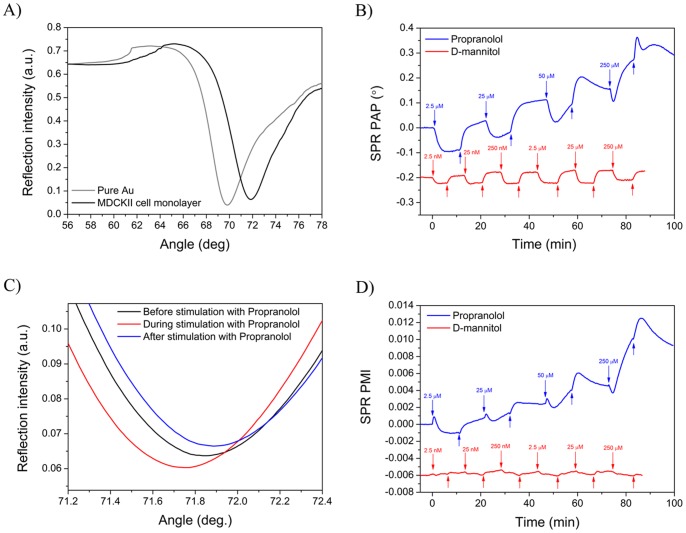
SPR signal responses during drug stimulation of MDCKII cells. A) Measured full SPR angular spectra of a pure gold coated SPR sensor slide (grey line) and MDCKII cell monolayer immobilized on the SPR sensor slide (black line). B) Measured changes in the angular position of the SPR peak minimum as a function of time when a MDCKII cell monolayer was stimulated with propranolol (blue line) and D-mannitol (red line). C) Focused part of full SPR angular spectra showing the main SPR peak curves measured before (black line), during (red line) and after (blue line) stimulating a MDCKII cell monolayer with 25 µM propranolol. D) Measured changes in the SPR peak minimum intensity as a function of time when a MDCKII cell monolayer was stimulated with propranolol (blue line) and D-mannitol (red line). In figure C) and D) the downward arrows represent the time of sample injections, and upwards arrows represent the injection of buffer without sample.

The successful immobilization of MDCKII cell monolayers on the SPR sensor slides was verified before each interaction measurement with drug compounds by first measuring the full SPR angular spectrum of the cell monolayer. Figure 5A shows a typical full SPR angular spectrum measured for a MDCKII cell monolayer. The full SPR angular spectrum for an MDCKII cell monolayer shows large shifts in the main SPR peak angular position, in the main SPR peak minimum intensity and in the shape of the TIR region compared to a pure SPR sensor slide. This confirmed the presence of a MDCKII cell monolayer on the SPR sensor slide. The main SPR peak angular position for the MDCKII cell monolayer was at 71.35° and the TIR region had a smooth shape located at ∼64°. These were very close to the main SPR peak angular position of 71.85° and the TIR region location of ∼62° for a simulated full SPR angular spectrum for a cell monolayer (see Fig. 4). This actually implicates that the MDCKII cell monolayer forms a low density waveguide on the SPR sensor slide. The differences in the angular position of the TIR region between measured and simulated full SPR angular spectra is a direct consequence of the fact that the simulated spectrum for a cell monolayer assumes a completely homogenous layer with a uniform thickness, whereas these assumptions are not necessarily valid for an actual living cell monolayer.

After verifying the quality of the MDCKII cell monolayer the MDCKII cells were stimulated with the test compounds and the actual full SPR angular spectra were measured as a function of time. Figure 5B shows a typical sensogram of the main SPR peak angular position for an experiment with confluent MDCKII cell monolayer when cells were exposed to increasing concentrations of propranolol and D-mannitol. The main SPR peak angular position for both compounds was displaced to lower angles during stimulation. However, when the cells were rinsed with pure running buffer the SPR angle started to increase and it took approximately 5 minutes for the baseline to stabilize. The main SPR peak angular position remained at higher values after stimulating the MDCKII cells with propranolol, whereas it returned to the baseline level after stimulation with D-mannitol. This suggests that a certain fraction of the propranolol remains in the cell monolayer after each stimulation, and that D-mannitol is almost completely removed from the cell monolayer after stimulation, regardless of the concentration used. The changes in the main SPR peak angular position were clearly concentration dependent for propranolol, but not for D-mannitol, for which the changes in the main SPR peak angular position were almost constant for all tested concentrations. These results indicate that propranolol and D-mannitol have different modes of interaction with the MDCKII cells which is reflected in the changes in the main SPR peak angular position. No significant changes in the main SPR peak angular position were measured when propranolol or D-mannitol was allowed to interact with a pure gold SPR sensor slide. This verifies that the main SPR peak angular position changes measured during interaction of the test compounds with the MDCKII cells actually reflect real drug-cell interactions and not drug-gold interactions.

The changes in the main SPR peak angular position when stimulating the MDCKII monolayer with propranolol and D-mannitol are surprisingly large, especially when considering the molecular weight of these compounds (i.e. 259.34 g/mol for propranolol and 182.17 g/mol for D-mannitol). This is in accordance with the simulated full SPR angular spectra for cell monolayers in Figure 4, where it was shown that very small changes in n or k in the cell monolayer induced rather large variations in the main SPR peak angular position and the main SPR peak minimum intensity.

Interestingly, when MDCKII cells were stimulated with propranolol it was found that not only the main SPR peak angular position changed during stimulation, but also the main SPR peak minimum intensity changed significantly (Fig. 5C–D and Video S1). In the case of D-mannitol only slight or no changes were observed in the main SPR peak minimum intensity during the MDCKII cell stimulation (Fig. 5D and Video S2), even though the main SPR peak angular position during cell stimulation with D-mannitol still showed a negative shift (Fig. 5B). These results further indicate that propranolol and D-mannitol have different modes of interaction with the MDCKII cells, which is reflected not only in the main SPR peak angular position but also in the main SPR peak minimum intensity. Thus, instead of analyzing only the main SPR peak angular position changes during drug-cell interactions it would be more useful to analyze both the main SPR peak angular position and the main SPR peak minimum intensity and plot these against each other in order to better understand and distinguish between the mode of interaction between different drugs during cell stimulation.

**Figure 6:**
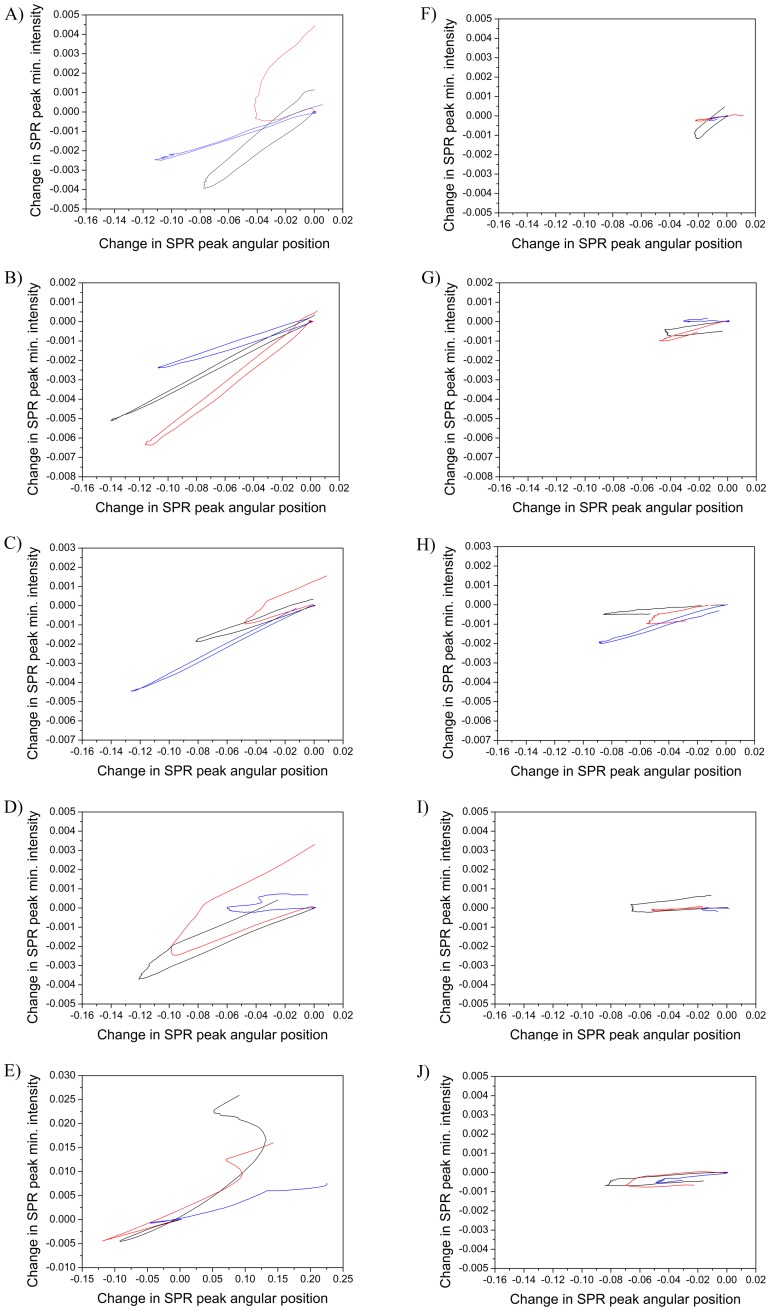
Change in SPR peak angular position versus minimum intensity during drug stimulation of MDCKII cells. Individual repetitions of the SPR measurements in a concentration series from three repetitions when MDCKII cell monolayers were stimulated with propranolol and D-mannitol, respectively: 2.5 nM (A: propranolol, F: D-mannitol), 250 nM (B: propranolol, G: D-mannitol), 2.5 μM (C: propranolol, H: D-mannitol), 25 μM (D: propranolol, I: D-mannitol) and 250 μM (E: propranolol, J: D-mannitol).

Figure 6 shows a series of plots of changes in the main SPR peak angular position versus main SPR peak minimum intensity from at least three repetitions and for several concentrations when MDCKII cells were stimulated with propranolol and D-mannitol. The difference between the behavior of the two compounds in terms of angle and intensity is very clear. Propranolol shows large changes in both angle and intensity which result in curves with significant slopes. D-mannitol on the other hand shows in most cases very small changes in intensity, leading to curves with a more horizontal appearance in these plots. The same trend in the intensity versus angle plots is repeated in the same way for each compound at all the concentrations tested.

The simulated full SPR angular spectra for a cell monolayer suggest that the TIR region should also reflect changes in the cell monolayer during drug stimulation (see Fig. 4C–E). When examining the TIR region in more detail during drug stimulation of MDCKII cells we found that the TIR angular position followed the same trend as the main SPR peak angular position when stimulating the MDCKII cells with propranolol or D-mannitol, i.e. larger changes for propranolol compared to D-mannitol (Figures S3, Figure S4A, Video S3 and Video S4). On the contrary, the reflection intensity of the TIR angular position (∼64°) showed a reverse trend compared to the main SPR peak minimum intensity, i.e. significant positive changes for propranolol and very small positive changes for D-mannitol (Figures S3, Figure S4, Video S3 and Video S4). These results are in sound accordance with the behavior of a cell monolayer described by the simulated full SPR angular spectra in Figure 4, where it was shown that the TIR angle and the main SPR peak angular position changes in the same direction with changes in *n*, and the reflection intensity in the TIR region changes in the opposite direction than the main SPR peak minimum with changes in *k*. Finally, when the TIR angular position was plotted against the reflection intensity of the TIR angular position, we found that the curves displayed similar slopes for both propranolol and D-mannitol, but the magnitude of the curve was clearly larger for propranolol than for D-mannitol (Figure S4C).

## Discussion

In this study, there were several reasons for using MDCKII cells and the two passively absorbing drugs utilizing different absorption routes. We anticipated at the beginning of our studies that the SPR signal responses with cells would be very complex if all kinds of drug transport processes were present. Therefore, the aim was to simplify the experimental design as much as possible by choosing a combination of cells and drugs which are already widely used specifically for passive (para- and transcellular) drug permeation studies. The choice of MDCKII cells was mainly based on the fact that they have low expression of drug transporters and little metabolic activity, which makes it a valuable cell line, especially for studying passive drug transport processes [36], [37]. D-mannitol and propranolol were chosen as model drugs because they are known to utilize different passive drug absorption routes, i.e. propranolol uses the transcellular and D-mannitol uses the paracellular absorption route [38], [39]. With this we could ensure that no other competing drug absorption processes, i.e. active transport and efflux of drugs, were present during our studies. In this way we could concentrate on studying and distinguishing the differences in the SPR signal responses which were solely due to passive drug absorption processes, without the interference from multiple competing processes.

Several studies have shown that the surface coverage of cells on the gold SPR sensor slide has a dramatic influence of the shape of the full SPR angle curve [12], [16], [24]. Therefore, in order to obtain repeatable SPR analyses and avoid unspecific interactions of the test compounds, not originating from cell interactions; it is of utmost importance to find a cell immobilization protocol that enables a repeatable preparation of confluent monolayers on the SPR sensor slide. In this study we found that MDCKII cells adhered and proliferated on gold and formed a confluent cell monolayer on the bare gold SPR sensor slide (Figure 2). We also found that no cell adhesion promoter was needed to form confluent monolayers of MDCKII cells on gold. Furthermore, Trypan blue tests showed that the gold SPR sensor slide surface is not cytotoxic for MDCKII cells (Figure 2F).

After reaching confluency, the MDCKII cells started to form clusters on the smooth gold SPR sensor slide surface. By examining the microscopy images it seemed that liquid had gathered under the cells at the clustering points. Cells are generally attached to surfaces in focal, close, and extracellular matrix contacts, each with its own characteristic separation distance from the surface [40]. As a result, cell plasma membranes are normally 10–100 nm away from the substrate surface depending on cell types and culturing conditions. However, due to the cluster formation in the case of MDCKII cells in this study the plasma membranes could have been even further away in those zones, which probably have a small effect on the quality of the measured full SPR angle spectrum of the MDCKII cell layer. This could explain why the small waveguide node appearing in the simulated full SPR angle spectra for a cell monolayer (Figure 4) is not properly visible in the measured full SPR angle spectrum of the MDCK II cells (Figure 5A).

Other cells than MDCKII have previously also been cultured on bare gold for SPR cell studies. These studies include cell lines such as human melanoma [22], human basophilic KU812 [41], RBL-2H3 rat mast and PAM212 mouse keratinocyte cells [19]. Moreover, Robelek and Weger [13] have grown MDCKII cells directly on SPR sensor surfaces as a confluent monolayer to study volume changes of cells. Based on the literature and the results in this study it seems that there is no general coating procedure or protocol for cell adhesion and preparation of confluent cell monolayers on gold coated SPR sensor slides, and therefore it is necessary to optimize these conditions for each cell line separately before performing SPR interaction measurements.

According to the full SPR angular spectra simulations a cell monolayer should form a low density waveguide with the main fundamental SPR peak minimum visible at relatively small angles compared to the thickness of the system (Figure 4). Additionally, the next node(s) remain close to the total internal reflection region, never progressing far from there. The actual full SPR angular spectrum measured for a confluent MDCKII cell layer (Figure 5A) resembled the simulated spectrum for a cell monolayer surprisingly well (Figure 4). This suggests that a monitoring of the shape of the full SPR angular spectrum thus provides a means to verify cell monolayer integrity during SPR interaction measurements. The full SPR angular spectra simulations also revealed that the sensitivity towards optical changes in a cell monolayer is enhanced by the fact that the cell covers the complete SPR evanescent field region, which enables the detection of extremely small changes in the living cell monolayer during cell stimulation. This has already been experimentally shown and indicated by other studies [15], [19], [20], but no studies have so far attempted to give an explanation of the implications of this on the full SPR angular spectra and how this could possibly be further utilized for living cell sensing with SPR.

The literature of SPR interaction measurements involving immobilized cells on the sensor surface are quite contradictory – in some cases the injection of the analyte results in positive SPR responses [15], [19], [20], [24], [26] and in other cases in negative SPR responses [18]–[21]. A widely accepted simplification for the measured SPR signal is that the main SPR peak angular position (or the intensity change at a fixed angle) is linearly proportional to the mass change in the evanescent field. This is also evident from the basic physics of the SPR phenomena [27] and the simulations in Figures 3A and 4B. Based on this it has been suggested that the SPR responses with cells based on measuring the changes in the main SPR peak angular position (or the intensity change at a fixed angle) originates from mass redistribution within the cells [19]. While possible and even probable, such a mass redistribution in cells should lead into changes **both** in the refractive index, **and** in the apparent light absorption of cell layers as different cell organelles and structures shift within the cell. This mass distribution could indeed induce either negative or positive changes in the SPR responses measured by monitoring only the main SPR peak angular position (or the intensity change at a fixed angle), depending on if the cytoskeletal mass migration in the cells is in the direction away from or towards the SPR evanescent field region during cell stimulation. This is also actually implicated by the simulations in Figure 3 and 4, and supported by the studies by Cuerrier et al., Chabot et al. [18], [21] and Yashunsky et al. [12]. Cuerrier et al. and Chabot et al. showed that morphological changes in cells, i.e. contraction of cells, induce a negative SPR shift in the reflection intensity measured at a fixed angle, while Yashunsky et al. showed that cell spreading induces an increase in the reflection intensity measured at a fixed angle with mid infra-red SPR.

However, none of the studies mentioned above have considered the changes in the main SPR peak angular position together with changes in the main SPR peak minimum intensity. It is clear from the simulated spectra in this study that changes ***both*** in the main SPR peak angular position ***and*** in the main SPR peak minimum intensity, and preferably also changes in the TIR region should be simultaneously monitored in order to fully understand the origin of the SPR responses caused by changes in the whole cell monolayer during drug stimulation of cells. This is because an increase/decrease in *n* will increase/decrease the main SPR peak angular position (Figure 4B) about twice as much as the TIR angle position (Figure 4D). Furthermore, an increase/decrease in *k* will be reflected in an increase/decrease in the main SPR peak minimum intensity and mainly a decrease/increase in the intensity around the TIR (Figure 4C and Figure 4E).

Often small molecular drugs or other compounds used for stimulating cells do not have significant absorptive properties in the wavelengths used in SPR devices and should generally cause a positive SPR response when measuring only the main SPR peak angular position (or the intensity change at a fixed angle). Thus, the simulations in Figure 4 suggest that a utilization of multiple parameters extracted from the full SPR angular spectra measured in real-time during cell stimulation would provide a means to determine the origin of the SPR response. In other words, this could provide a way to distinguish whether the SPR response 1) originates only from the accumulation of the stimulant in the cell layer, 2) is due to morphological changes of the cells or cytoskeleton mass redistribution within the cells, or 3) is a combined effect of 1) and 2). The plots of the main SPR peak minimum intensity versus the main SPR peak angular position in Figure 6 and the changes in the TIR region (Figures S3 and Figure S4) actually highlight that there is a clear difference in the interaction modes of propranolol and D-mannitol with the MDCKII cells. It is known that propranolol utilizes the transcellular pathway and D-mannitol utilizes the paracellular pathway when absorbed through a cell monolayer [38], [39]. The graphical representation in Figure 6 in combination with the changes in the TIR region (Figure S4C) thus provides the means to differentiate between the modes of action of drugs with cells.

When examining Figure 5B, Figure 5D and Figure 6A–E in more detail it is obvious that there is a clear negative shift in both the SPR peak angular position and the SPR peak minimum intensity during cell stimulation with propranolol when examining each concentration separately. After stimulation, both the main SPR peak angular position and the main SPR peak minimum intensity return to a higher level than before stimulation. This indicates that some propranolol accumulates in the cells as expected because of its ability to diffuse transcellularly into cells. It is worthwhile to note from Figure 5B, Figure 5D and Figure 6E that the change in both the main SPR peak angular position and the main SPR peak minimum intensity quickly becomes positive during stimulation of the MDCKII cell with the highest concentration for propranolol (i.e. 250 μM). These results for propranolol indicate that a stimulation of the cells with a low concentration of propranolol first induces a cell contraction accompanied by a mass redistribution away from the evanescent field region leading to a negative shift in both of the SPR responses. Hence, the SPR responses for low concentrations of propranolol probably originate from morphological and mass distribution changes of the cells, rather than from propranolol absorbed into the cell, since the amount of absorbed propranolol is not sufficient to render the SPR signals positive. A stimulation of the MDCKII cells with the highest concentration of propranolol causes an initial negative shift in both SPR responses. This suggests that a stimulation with propranolol first induces cell contraction accompanied by a mass redistribution away from the evanescent field region. Hereafter, both SPR responses become positive, which is probably a consequence of a cell spreading accompanied by a mass redistribution towards the evanescent field region and an accumulation of propranolol in the cells. Similar behavior has been shown for HEK-293 cells stimulated with angiotensin II by correlating the change in the SPR intensity at a fixed angle with phase-contrast microscopy imaging [18].

In the case of D-mannitol (Figure 5B, Figure 5D and Figure 6F–J), a clear negative shift is only seen in the angular position of the SPR peak minimum during cell stimulation, while the SPR peak minimum intensity only changes very little or not at all. After cell stimulation, the main SPR peak angular position returns to the same level as before stimulation. This could be expected because D-mannitol diffuses through cell layers by the paracellular pathway, and no accumulation of D-mannitol is expected in the cell monolayer or within the cells. However, it was surprising to see that the SPR peak angular position during cell stimulation with D-mannitol showed a negative shift even though there was no change in the main SPR peak minimum intensity. This could indicate that D-mannitol indeed induces a cell contraction in order to utilize the paracellular pathway, but the contraction is not sufficiently large to induce any cell mass redistribution within the cell. This perception is also supported by the fact that the changes in the main SPR peak angular position are basically constant for all concentrations and much smaller in the case of D-mannitol compared to propranolol (Figure 5B and Figure 6). Furthermore, the smaller changes in the TIR region (Figure S3 and Figure S4) indicate a smaller mass redistribution within the cells in the case of D-mannitol compared to propranolol. This is also supported by the simulations in Figure 4C–E, which suggest that the contribution to the TIR region origins mainly from the cell monolayer outside the evanescent field and does not have any significant contributions from morphological changes of the cells. This could mean that the TIR region merely reflects the mass redistribution within the cell monolayer.

The results from our study presented here convinced us that the change in the main SPR peak angular position reflects both drug accumulation and morphological changes in the cell monolayer, and that the change in the main SPR peak minimum intensity is mainly due to mass redistribution within the cells. Thus, based on the results in this work, we suggest the following two possible scenarios for the SPR responses in living cell sensing: 1) An accumulation of a drug, e.g. D-mannitol, in the cell layer which induces only a slight contraction of the cells but no mass redistribution in the cells, would result in a negative change in the SPR peak angular position, but it would not result in a change in the SPR peak minimum intensity. 2) An accumulation of a drug, e.g. propranolol, in the cell layer which induces a contraction of the cell layer and a mass redistribution in the cells, would result in a negative change in both the SPR peak angular position and the SPR peak minimum intensity. If taking into account all other possible options, there could be two additional scenarios for the SPR responses in living cell sensing which could not be addressed by the experimental design in this work. Namely, 1) a simple accumulation of a drug in the cell layer which does not induce any morphological changes in the cell layer, would result in a positive change in the SPR peak angular position, but no change in the SPR peak minimum intensity, and 2) an accumulation of a drug in the cell layer which induces a spreading of the cell layer, and a mass redistribution in the cells would result in a positive change in both the SPR peak angular position and the SPR peak minimum intensity.

Conclusively, it is rather obvious that the origins of the SPR responses are very complex when combining SPR with living cells. In this work we have presented an attempt to better understand the SPR responses in living cell sensing by comparing optical modeling of cell monolayers with multiple parameters extracted from full angular SPR spectra recorded in real-time during cell stimulation with two model drugs. Simulated full SPR angular spectra in combination with changes in the main SPR peak angular position, the main SPR peak minimum intensity, and changes in the TIR region indicated that the change in the main SPR peak angular position, traditionally used in SPR studies, does probably not only reflect simple mass accumulation or mass redistribution within the cells. Instead, it probably also reflects morphological changes in the cell layer, which actually could dominate the main SPR peak angular position response. The change in the main SPR peak minimum intensity, on the other hand, is suggested to reflect which type of change in the cell layer (morphological and/or mass redistribution) causes the changes in the main SPR peak angular position.

Finally, plotting the main SPR peak angular position versus the main SPR peak minimum intensity showed specific signal patterns. These signal patterns are suggested to reflect the type of drug absorption route utilized by the drug. We believe that the results in this study provide a step forward to an improved understanding of the signal responses in living cell sensing with SPR. This can open up new opportunities for utilizing the SPR technology in a broader context in the field of life sciences, for example as a tool for providing real-time complementary information for traditional *in vitro* cell assays in order to obtain a better mechanistic understanding of drug-cell interactions on a cellular level.

## Supporting Information

Figure S1 **Simulated full SPR angular spectra demonstrating that very large changes in the complete cell monolayer thickness (** ***d_ef_ + d_cell_*** **) do not affect the main SPR peak position.** It is worth noting that the complete cell monolayer thickness has to change dramatically before it induces any significant changes in the shape of the TIR region. The following parameters were used for simulations: *n_ef_*  =  *n_cell_*  = 1.34, *k_ef_*  =  *k_cell_*  = 0.002 and *n_bulk_*  = 1.3299, *k_bulk_*  = 0.
(TIF)

Figure S2 **Simulated full SPR angular spectra demonstrating that changes in** ***n_bulk_*** **of the bulk medium layer above the cell monolayer do not affect the main SPR peak and induce only very small changes in the shape of the TIR region.** The following parameters were used for simulations: *n_ef_*  =  *n_cell_*  = 1.34, *k_ef_*  =  *k_cell_*  = 0.002 and *k_bulk_*  = 0.
(TIF)

Figure S3 **Measured full SPR angular spectra at selected time points after starting the stimulation of a MDCKII cell monolayer with A) 25** **μM D-mannitol and B) 25** **μM propranolol.** Th time points for recording the full SPR angular spectra in A) were t  = 0 min (black solid line), t = 2 min (red solid line), t = 4 min (blue solid line), t = 13 min (black dashed line), and in B) t = 0 min (black solid line), t = 2 min (red solid line), t = 5 min (blue solid line), t = 17 min (black dashed line).
(TIF)

Figure S4 **A**) Change in the TIR angle position measured as a function of time during stimulation of a MDCKII cell monolayer with 25 μM Propranolol (blue line) or D-mannitol (red line). These results suggest that there is a much higher mass redistribution away from the cell monolayer region within the evanescent field (Fig. 4A, region III) for propranolol than for D-mannitol. B) Change in the intensity at TIR angle position measured as a function of time during a stimulation of a MDCKII cell monolayer with 25 μM Propranolol (blue line) or D-mannitol (red line). These results indicate that there is a much higher analyte accumulation and mass redistribution towards the cell monolayer region outside the evanescent field (Fig. 4A, region II) for propranolol than for D-mannitol. C) Change in the intensity at TIR angle position versus change in TIR angle position for 25 μM Propranolol (blue line) or D-mannitol (red line) during stimulation of a MDCKII cell monolayer. Note that the slopes of these curves are the same, while the magnitude is clearly different indicating that an overall larger mass redistribution within the cell monolayer takes place during stimulation with propranolol than with D-mannitol. The same slope of these curves strongly suggests that the TIR region of the full SPR angular spectrum actually merely reflects accumulation of analytes and mass redistribution within the cell monolayer, but does probably not have any contribution from the adhesion and contact area of the cells.
(TIF)

Video S1 **Change in the SPR peak angular position and SPR peak minimum intensity measured during a stimulation of a MDCKII cell monolayer with 25** **µM Propranolol (sample injection ∼4** **s, buffer injection ∼16** **s).** The video is a speed-up representation of a 24 minute-measurement.
(AVI)

Video S2 **Change in the SPR peak angular position and SPR peak minimum intensity measured during a stimulation of a MDCKII cell monolayer with 25** **µM D-mannitol (sample injection ∼5** **s, buffer injection ∼12** **s).** The video is a speed-up representation of a 16 minute-measurement.
(AVI)

Video S3 **Change in the TIR region measured during a stimulation of a MDCKII cell monolayer with 25** **µM Propranolol (sample injection ∼4** **s, buffer injection ∼14** **s).** The video is a speed-up representation of a 24 minute-measurement.
(AVI)

Video S4 **Change in the TIR region measured during a stimulation of a MDCKII cell monolayer with 25** **µM D-mannitol (sample injection ∼5** **s, buffer injection ∼13** **s).** The video is a speed-up representation of a 16 minute-measurement.
(AVI)
