# Supplementary figures and images for: Elucidating the Signal Responses of Multi-Parametric Surface Plasmon Resonance Living Cell Sensing: A Comparison between Optical Modeling and Drug–MDCKII Cell Interaction Measurements
Source: PLoS One. 2013 Aug 27;8(8):e72192. doi: 10.1371/journal.pone.0072192 (PMC3754984; doi:10.1371/journal.pone.0072192)

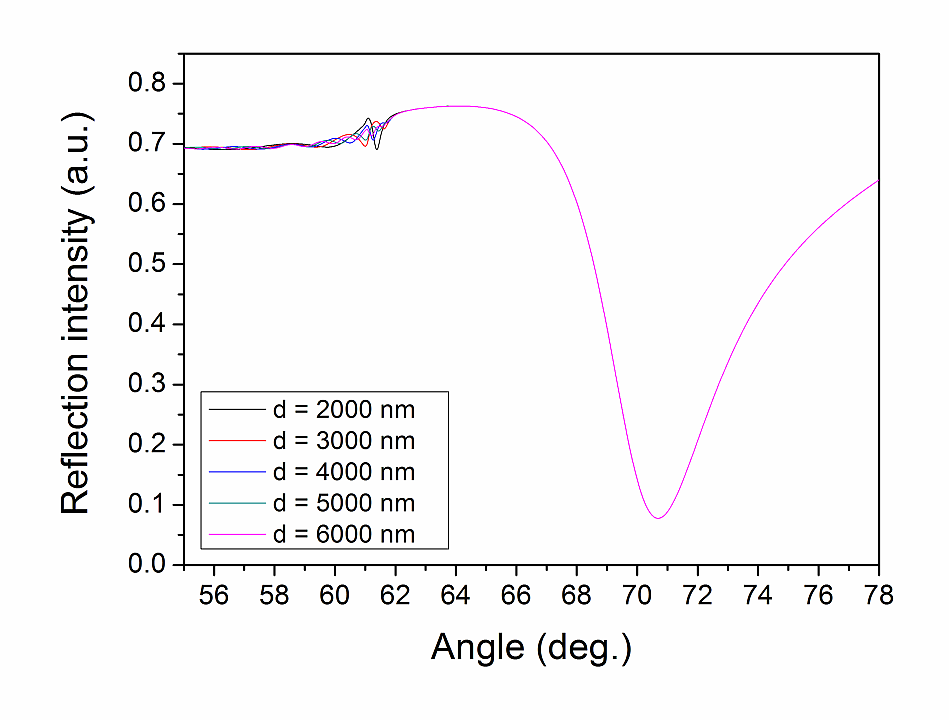

Supplement: Figure S1 — Simulated full SPR angular spectra demonstrating that very large changes in the complete cell monolayer thickness ( def + dcell ) do not affect the main SPR peak position. It is worth noting that the complete cell monolayer thickness has to change dramatically before it induces any significant changes in the shape of the TIR region. The following parameters were used for simulations: nef = ncell = 1.34, kef = kcell = 0.002 and nbulk = 1.3299, kbulk = 0. (TIF) [file pone.0072192.s001.tif]

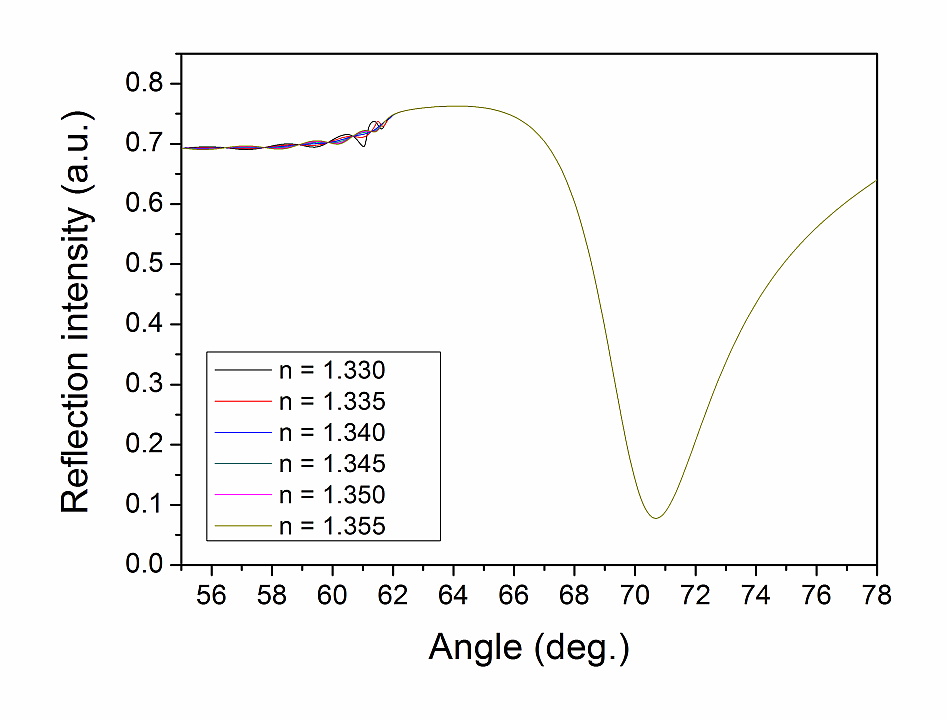

Supplement: Figure S2 — Simulated full SPR angular spectra demonstrating that changes in nbulk of the bulk medium layer above the cell monolayer do not affect the main SPR peak and induce only very small changes in the shape of the TIR region. The following parameters were used for simulations: nef = ncell = 1.34, kef = kcell = 0.002 and kbulk = 0. (TIF) [file pone.0072192.s002.tif]

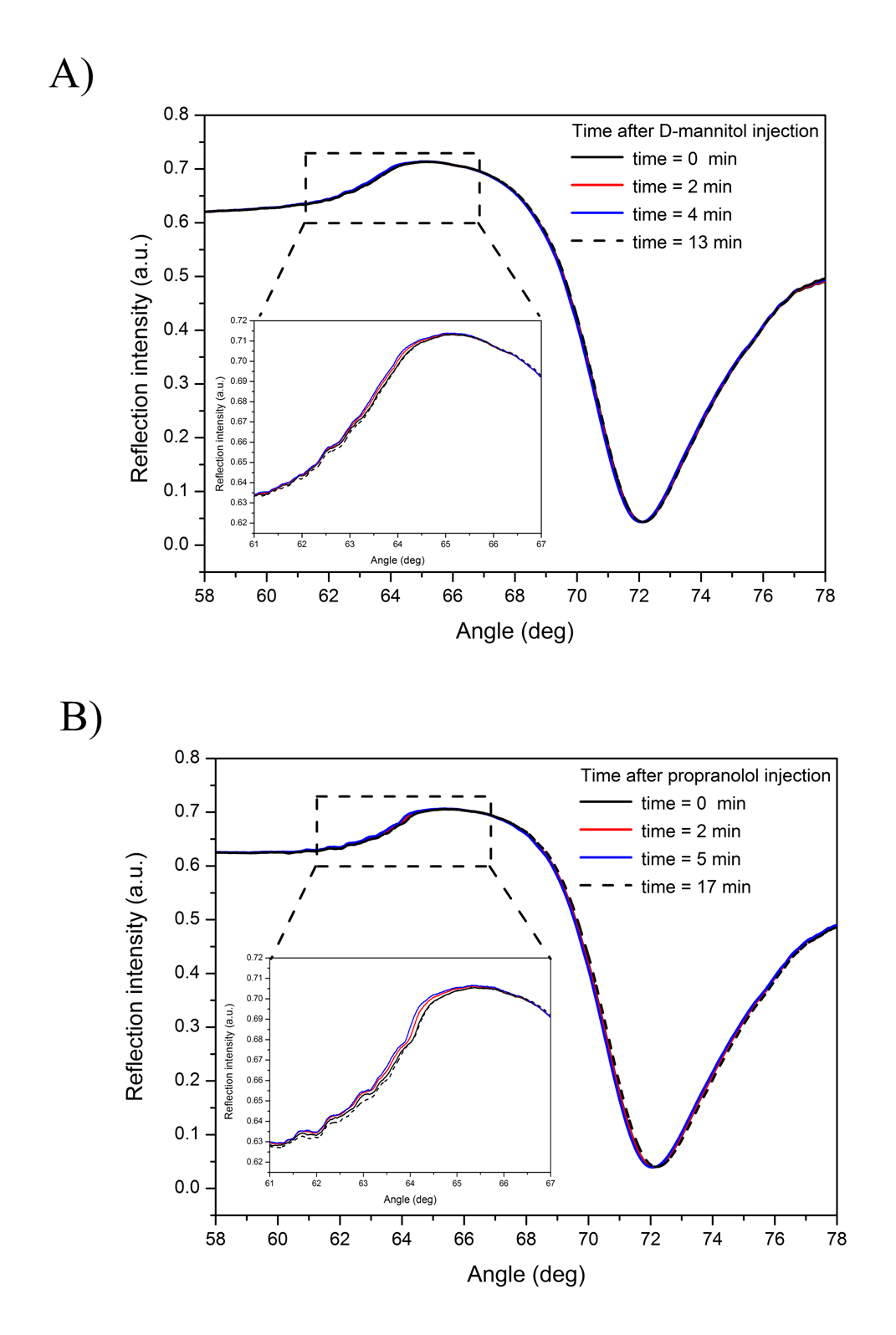

Supplement: Figure S3 — Measured full SPR angular spectra at selected time points after starting the stimulation of a MDCKII cell monolayer with A) 25 μM D-mannitol and B) 25 μM propranolol. Th time points for recording the full SPR angular spectra in A) were t = 0 min (black solid line), t = 2 min (red solid line), t = 4 min (blue solid line), t = 13 min (black dashed line), and in B) t = 0 min (black solid line), t = 2 min (red solid line), t = 5 min (blue solid line), t = 17 min (black dashed line). (TIF) [file pone.0072192.s003.tif]

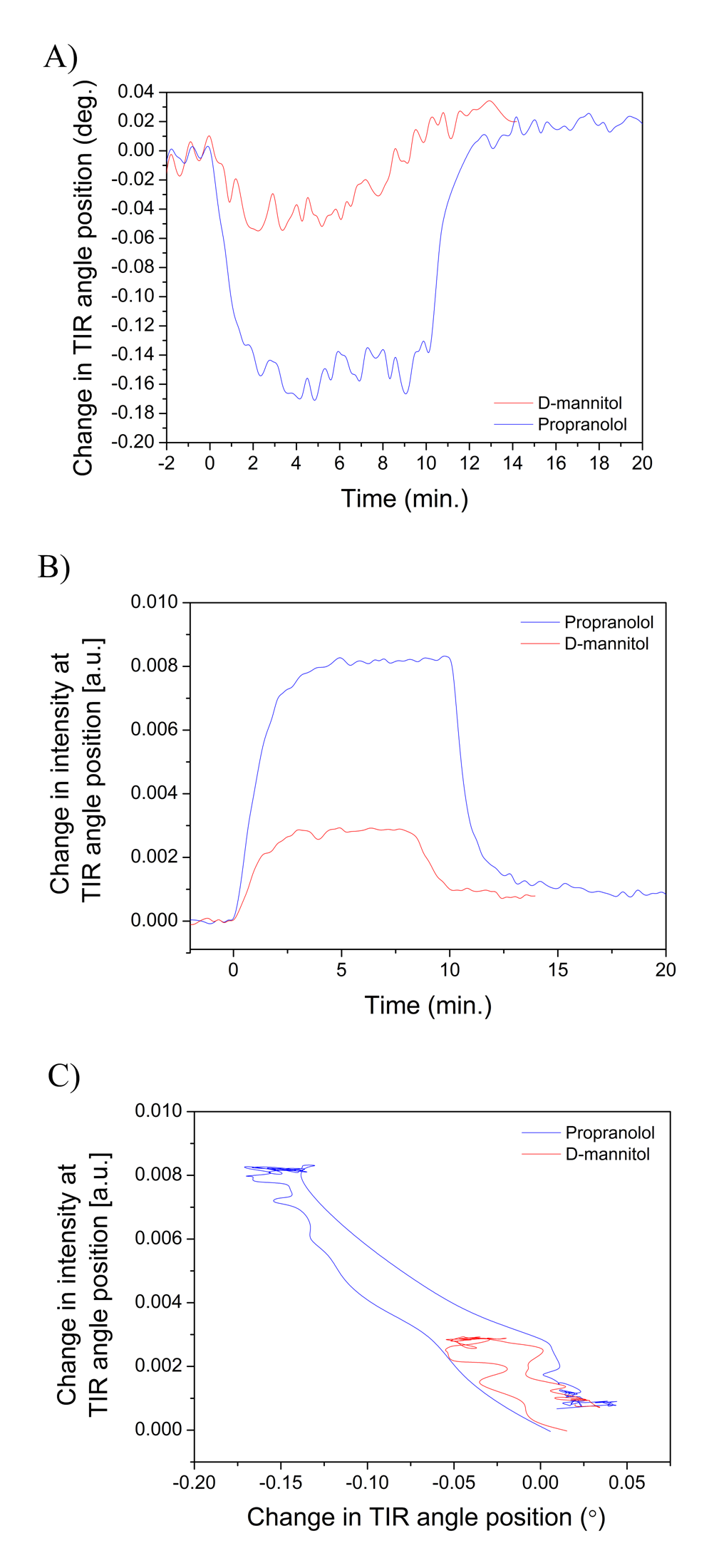

Supplement: Figure S4 — A) Change in the TIR angle position measured as a function of time during stimulation of a MDCKII cell monolayer with 25 μM Propranolol (blue line) or D-mannitol (red line). These results suggest that there is a much higher mass redistribution away from the cell monolayer region within the evanescent field (Fig. 4A, region III) for propranolol than for D-mannitol. B) Change in the intensity at TIR angle position measured as a function of time during a stimulation of a MDCKII cell monolayer with 25 μM Propranolol (blue line) or D-mannitol (red line). These results indicate that there is a much higher analyte accumulation and mass redistribution towards the cell monolayer region outside the evanescent field (Fig. 4A, region II) for propranolol than for D-mannitol. C) Change in the intensity at TIR angle position versus change in TIR angle position for 25 μM Propranolol (blue line) or D-mannitol (red line) during stimulation of a MDCKII cell monolayer. Note that the slopes of these curves are the same, while the magnitude is clearly different indicating that an overall larger mass redistribution within the cell monolayer takes place during stimulation with propranolol than with D-mannitol. The same slope of these curves strongly suggests that the TIR region of the full SPR angular spectrum actually merely reflects accumulation of analytes and mass redistribution within the cell monolayer, but does probably not have any contribution from the adhesion and contact area of the cells. (TIF) [file pone.0072192.s004.tif]
